# The Antibacterial and Cytotoxic Effects of Silver Nanoparticles Coated Titanium Implants: A Narrative Review

**DOI:** 10.3390/ma15145025

**Published:** 2022-07-19

**Authors:** Håvard J. Haugen, Soukayna Makhtari, Sara Ahmadi, Badra Hussain

**Affiliations:** 1 Department of Biomaterials, Institute of Clinical Dentistry, University of Oslo, 0317 Oslo, Norway; badra.hussain@odont.uio.no; 2 Institute of Clinical Dentistry, University of Oslo, 0317 Oslo, Norway; soukaym@student.odont.uio.no (S.M.); saraahm@student.odont.uio.no (S.A.)

**Keywords:** silver nanoparticles, antibacterial, dental implants, implant surface modification

## Abstract

Nanotechnology has become an emerging research field with numerous biomedical scientific applications. Silver possesses bactericidal activities that have been harnessed for centuries; however, there is a concern about the toxic effects of silver nanoparticles. This paper aims to provide an overview of silver-treated dental implants and discuss their potential to reduce the prevalence of peri-implant diseases. An electronic search was performed using PubMed. After screening, data extraction was performed on the 45 remaining articles using inclusion and exclusion criteria. Most of the articles demonstrated that silver nanoparticles embedded in a coating layer and/or on surface-treated titanium exhibit sound antibacterial effects and biocompatibility. Most of the reviewed studies revealed that silver nanoparticles on dental implant surfaces reduced cytotoxicity but provided a prolonged antibacterial effect. The cytotoxicity and antibacterial effect are closely linked to how the silver nanoparticles are released from the titanium surfaces, where a slower release increases cell viability and proliferation. However, to improve the clinical translation, there is still a need for more studies, especially evaluating the long-term systemic effects and studies recreating the conditions in the oral cavity.

## 1. Introduction

Nanotechnology has become an emerging research field with various applications in technology and science. According to the European Union (EU), nanoparticles are defined as “A natural, incidental or manufactured material containing particles, in an unbound state or as an aggregate or as an agglomerate and where, 50% or more of the particles in the number size distribution, one or more external dimensions is in size range 1–100 nm”. EU’s *Official Journal* on 20 October 2011. Structures in the nanometer range possess unique properties desirable in materials science and biology [1]. Notably, research on silver nanoparticles has been popular in recent decades [2]. Silver ions possess bactericidal activities that have been harnessed for centuries [3]. The chemical state of silver varies between the following forms, cationic silver (Ag^+^), metallic silver (Ag^0^) and silver oxide particulates, and depends on the immediate environment such as body fluids, aqueous biological media, etc. The chemical forms of silver can be transformed through chemical speciation, agglomeration and dissolution [4].

**Figure 1:**
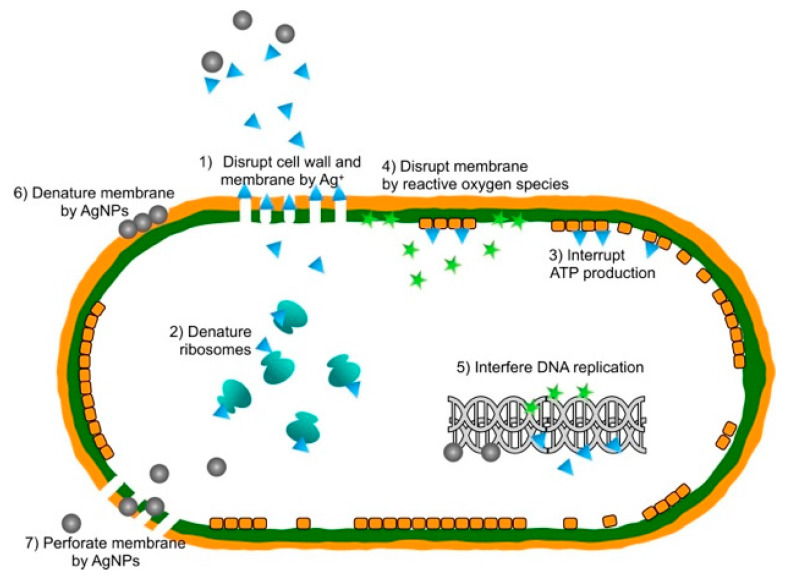
The antibacterial actions of silver nanoparticles (AgNPs). (1) Disruption of the cell wall and cytoplasmic membrane: silver ions (Ag^+^) released from silver nanoparticles adhere to or pass through the cell wall and cytoplasmic membrane. (2) Denaturation of ribosomes: silver ions denature ribosomes and inhibit protein synthesis. (3) Interruption of adenosine triphosphate (ATP) production is terminated because silver ions deactivate respiratory enzymes on the cytoplasmic membrane. (4) Membrane disruption by reactive oxygen species: reactive oxygen species produced by the broken electron transport chain can cause membrane disruption. (5) Interference of deoxyribonucleic acid (DNA) replication: silver and reactive oxygen species bind to deoxyribonucleic acid and prevent replication and cell multiplication. (6) Denaturation of membrane: silver nanoparticles accumulate in the cell wall pits and cause membrane denaturation. (7) Perforation of membrane: silver nanoparticles directly move across the cytoplasmic membrane, which can release organelles from the cell. Reprinted with permission from [14]. Originally published by and used with permission from Dove Medical Press Ltd. Copyright remains with the author and Dove Medical Press Limited, no transfer of copyright is inferred or implied.

**Figure 2:**
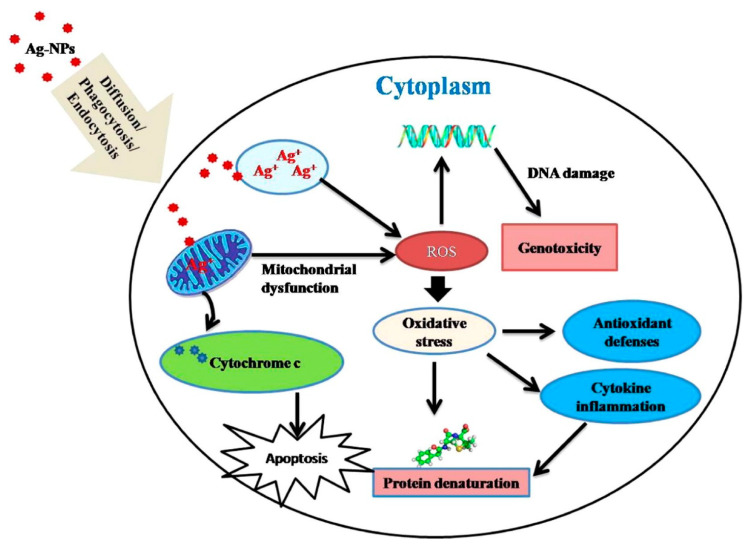
The possible uptake process and mechanism of cytotoxicity induced by Ag-NPs in different cell lines are based on several studies’ metadata. Reprinted with permission according to license CC BY-NC-ND 4.0 [18].

Silver nanoparticles (AgNP) are nanoparticles of silver with a diameter of less than 100 nm. However, they do not have to exhibit a spherical shape., The mechanism of the antimicrobial action of silver is highly dependent on the morphology (size and shape) of Ag particles [5,6], e.g., Raza et al. noticed that the smallest-sized spherical AgNPs showed higher microbial activity against *Pseudomonas aeruginosa* and *Escherichia coli* when compared to the triangular and larger spherical shaped AgNPs. Due to their nanoscale size has a large surface-to-volume ratio and can exhibit remarkable bactericidal activity even at low concentrations [7]. The silver nanoparticles’ antibacterial mechanism is illustrated in Figure 1. Silver nanoparticles are used in biomedical and therapeutical applications such as molecular diagnostics, medical imaging, drug delivery, surgical mesh, wound dressings, artificial joint replacements, and medicaments for wound healing promotion [8,9] Silver nanoparticles can be manufactured in various routes [10,11,12]. Helmlinger et al. investigated the influence of silver nanoparticle morphology on dissolution kinetics and its effect on cells and bacteria. They found silver nanoparticles of different sizes and shapes: with equal cytotoxicity but different antibacterial effects. The antibacterial effect increased with a higher dissolution rate, which was explained due to dissolved silver ions (Figure 2) [13].

In dentistry, researchers are trying to harness the antibacterial properties of silver nanoparticles by incorporating them into different materials used in the field, such as composite resin for direct restorations, adhesive materials, acrylic resins used in the fabrication of dentures, guided tissue regeneration membranes, irrigation and obturation materials in endodontics. This article will focus on using silver nanoparticles on titanium dental implants and their potential to reduce the prevalence of peri-implant diseases [15] However, the clinical use of nano-Ag is limited, primarily because of its potential adverse effects. It has been demonstrated that silver nanoparticles were directly associated with the number of free silver ions released in the medium [16,17].

There is a concern that silver nanoparticles may act as a Trojan horse, entering cells and releasing silver ions which may interfere with cell functions [19]. Some laboratory studies have reported that AgNPs may induce oxidative stress and impair mitochondrial function in human cells [20]. In addition, several vitro studies indicate that AgNPs are cytotoxic to mammalian cells derived from skin, brain, liver, lung, reproductive, vascular, and vascular systems [21]. Numerous studies have shown that some metallic nanoparticles may induce decreased cell survival rate, production of reactive oxygen species, mitochondrial damage, DNA strand breaks, and even autophagy, pyroptosis, apoptosis, or other forms of cell death [22]. However, a study on Labradors indicated that AgNPs had excellent biocompatibility and enhanced bone formation around dental implants [23]. So far, the data is inconclusive; therefore, more studies assessing in vitro toxicity and long-term in vivo studies focusing on mutagenicity and cytotoxicity should be carried out to ensure the clinical safety of these particles. According to the 1st European workshop on periodontology, peri-implantitis is defined as “an inflammatory process affecting the tissues around an osseointegrated implant in function, resulting in loss of supporting bone” and peri-implant mucositis is defined as a reversible inflammation of the mucosa surrounding a functional implant [24]. It is hypothesized that the onset of peri-implant diseases is caused by an imbalance between the host defence and the bacterial load [25]. After the implantation in the oral cavity, the transmucosal abutment of the implant will immediately be coated with an acquired pellicle, promoting the attachment of oral bacteria at the abutment surface and initiating biofilm formation [26]. Biofilms resist host defence and antimicrobial agents [27,28,29].

This review aimed to provide an overview of silver-treated dental implants and discuss their potential to combat peri-implantitis, a possible step to increase clinical translation of such surface coatings.

## 2. Literature Search Strategy

An electronic literature search was conducted in PubMed. In order to identify articles, the following terms were used “Silver nanoparticles AND dental implants NOT review”, “Silver nanoparticles AND (“dental implants or dental implant”) NOT review”, Silver nanoparticles AND (“dental implants” or “dental implant”) AND antibacterial NOT review, (“Dental implants” OR “dental implant”) AND (“Surface modifications” OR coating OR immobilized”) AND (“Antibacterial OR “bacterial adhesion” OR “antimicrobial”) AND (“silver nanoparticles” OR AgNP OR AgNPs OR silver”) NOT review.

### Study Selection Criteria

The titles and abstracts were screened according to the selection criteria. The inclusion and exclusion criteria implemented are presented below:

Inclusion criteria:

- Publication in English;
- Relation to implant dentistry;
- Year of publication (2010–2021);
- Original articles;
- Controlled trials with titanium;
- Focus on silver nanoparticles on dental implants;

Exclusion criteria:

- Case report, Conference paper, Reviews;
- Not related to dental implants;
- Not focused on silver nanoparticles;
- Inadequate control;
- No antibacterial test.

## 3. Literature Outcome

An electronic search in Pubmed identified a total of 211 articles. Screening titles and abstracts led to the exclusion of 156 articles, including duplicates automatically removed by PubMed, resulting in 55 remaining articles. After the analysis of full texts, ten articles were excluded. Further data extraction was performed on the 45 remaining studies (Table 1).

Different surface modifications and doping methods have been used to attach the silver nanoparticles to the titanium surfaces. The main application methods and surface modifications that will be discussed in this article include the use of linking components to embed silver nanoparticles on the titanium surface, glass coatings on titanium implants containing silver nanoparticles, penetration and implantation of silver nanoparticles in nano/microstructured titanium surfaces. An ideal dental implant surface is biocompatible with the implanted tissue that promotes osseointegration while preventing bacterial adhesion and biofilm formation, which may cause further development of peri-implant mucositis or peri-implantitis [30] This article examines how silver nanoparticles on titanium surfaces with different surface modifications influence antibacterial activity, biocompatibility/cytotoxicity, and osseointegration.

**Table 1:** Overview of available silver treatment for titanium dental implant.

Ag—Treatment on Ti-Surface	Coatings/Surface Modifications	Results	Author and Year
Ag-coating on Ti with a polymeric molecule	AgNP and gentamycin are embedded in a silk fibroin coating, and immobilized in the titanium surface using polydopamine.	A combination of silver nanoparticles (AgNPs) and a kind of antibiotic can synergistically inhibit bacterial growth, where a low concentration of AgNPs has been confirmed to promote the proliferation and osteogenesis of osteoblasts.	Wenhao Zhou et al., 2017 [31], in vitro study
	Titanium surface deposited with catechol-functionalized chitosan and AgNPs.	Exhibited good antibacterial activity, and minimal cytotoxicity towards L929 mouse fibroblast cells.	Cheng et al., 2019 [32], in vitro study
	Titanium surface primed with phase transited lysozyme with subsequent layers of hyaluronic acid and chitosan loaded with AgNPs.	Exhibited antibacterial activity, facilitated osseointegration.	Zhong et al., 2016 [33], in vitro study
	Compares silver nanoparticles embedded on to titanium coated with hydroxyapatite/chitosan biopolymer and titanium surface without the biopolymer coating.	Exhibited antibacterial activity.	Venugopal et al., 2017 [34], in vitro study
	Chemical vapour deposition was used to synthesize and apply to synthesize and apply the polysiloxane plasma polymer, and the metallic silver particles were deposited on the surface using physical vapour deposition. Polysiloxane layer on the titanium and over the silver nanoparticles.	The coated implants exhibited better osseointegration results when the surface was modified by acid-etching and grit-blasting.	Smeets et al., 2017 [35], in vitro study
	Titanium surface modified with polydopamine and AgNPs	Exhibited appropriate antimicrobial effect.	Choi et al., 2019 [36], in vitro study
	Implants coated with Poly-L-Lysine/Sodium Alginate and AgNPs.	Exhibited antibacterial activity, and prevented bacterial adhesion and colonization. Facilitated mineralization on the surface.	Guo et al., 2020 [37], in vitro study
	Titanium disc coated with an acrylate-based photocatalytic copolymer that embeds AgNPs.	Exhibited antibacterial activity.	Gyorgyey et al., 2016 [38], in vitro study
	Plasma chemical oxidized titanium surface coated with AgNPs and ionic zinc.	Coating with zinc exhibited a longer duration of antibacterial activity compared with Ag-coating. However, Ag-coating showed better biocompatibility.	Kranz et al., 2019 [39], in vitro study
	Titanium nanotubes coated with polydopamine-induced nanocomposite silver/calcium phosphate.	Exhibited good antibacterial effect and in vitro cytocompatibility to MG63 cells.	Li et al., 2019 [40], in vitro study
Glass containing Ag-NPS	Titanium surface coated with soda-lime glass containing AgNPs	Inhibited biofilm formation of in vitro Streptococcus oralis.	Cabal et al., 2012 [41], in vitro study
	Titanium coated with highly ordered nanoporous silica and AgNPs.	Reduced bacterial adhesion and biofilm formation.	Massa et al., 2014 [42], in vitro study
	Titanium abutments coated with soda-lime-glass/nAg-powder. A clinical study was performed in Beagle dogs.	Reduced bone loss in experimental peri-implantitis.	Martinez et al., 2014 [43], in vivo study
	Titanium abutments coated with soda-lime glass/Ag powder, a study in the Beagle dog.	Reduced bone loss in experimental peri-implantitis.	Lopez et al., 2012 [44], in vivo study
Penetration of AgNPs in nanotubes	Titanium nanotubes immersed in AgNO_3_ and AgNO_3_ followed by immersion in glucose.	Exhibited bacteriostatic rate at 99.99%, low toxicity and high osteogenetic potential.	Wang et al., 2013 [45], in vivo study
	Titanium Nanotubes modified with basic fibroblast growth factor cross-linked with polydopamine, then immersed in AgNO_3_.	Exhibited bacterial inhibition, reduced pro-inflammatory factors. Enhanced osteogenic differentiation of dental pulp stem cells.	Albashari et al., 2021 [46], in vitro study
	Titanium nanotubes hydrothermally treaded to incorporate Sr, then immersed in AgNO_3_ solution.	Exhibited long-lasting antibacterial effect and inhibited bacterial adhesion. With no evident cytotoxic effects. Showed improved repair of cortical bone repair and increased trabecular bone microarchitecture in rat tibia.	Cheng et al., 2016 [47], in vivo study
	Titanium nanotubes coated by magnetron-sputtering of Ag-ions.	Exhibited antibacterial activity, low toxicity depending on different sputtering time. The 60 s sputtering time showed minimal toxicity.	Uhm et al., 2014 [48], in vitro study
	Titanium nanotubes coated by magnetron sputtering technique to deposit Ag or Zn.	Exhibited antimicrobial activity and inhibited microbial adhesion.	Roguska et al., 2018 [49], in vitro study
	Titanium nanotubes are coated by polydopamine and used as a linking component to add AgNPs.	Exhibited antibacterial activity, and inhibited bacterial adhesion. Showed cell toxicity on HGF and U2OS cells.	Ren et al., 2021 [50], in vitro study
	Titanium nanotubes coated with nanosized or microsized silver reduced by δ-gluconolactone.	Nanosized clusters exhibited slower silver release and better antibacterial activity.	Gunputh et al., 2018 [51], in vitro study
	Titanium nanotubes coated with silver are reduced by δ-gluconolactone with or without a topcoat of nano hydroxyapatite.	The addition of nano hydroxyapatite did not compromise the antibacterial activity.	Gunputh et al., 2020 [52], in vitro study
	Titanium nanotubes coated with Ag/CaP using polydopamin as a linking component.	Exhibited antibacterial activity, relatively good cytocompatibility to MG63 cells and enhanced osteogenic differentiation.	Li et al., 2015 [53], in vitro study
	AgNPs/FGF-2 immobilised in nanotubular titanium surface.	Exhibited cytocompatibility, negligible cytotoxicity and improved cell functions such as ECM-related gene expression, cell attachment and proliferation.	Ma et al., 2011 [54], in vitro study
Titanium surface modified using Ag-PIII	SLA-treated titanium modified using silver plasma immersion ion implantation.	Exhibited antibacterial activity and no apparent toxicity towards rBMSC cells.	Zhu et al., 2015 [55], in vitro study
	SLA-treated titanium modified using silver plasma immersion ion implantation. Canine in vivo study s.	Enhanced new bone formation, bone mineral density and trabecular pattern. Ti-AgPIII for 30 min showed better osteogenic indicators compared to the 60 and 90 min samples.	Qiao et al., 2015 [23], in vivo study
	Titanium surface modified with Zn/Ag by using plasma immersion ion implantation.	Exhibited antibacterial and osteogenic activity.	Jin G et al., 2014 [56], in vitro study
Plasma electrolytic oxidation (PEO)	AgNP immobilized on rationally designed, and selective lasered porous titanium implants using plasma electrolytic oxidation.	Exhibited antibacterial activity without any signs of cytotoxicity on hMSCs.	Van Hengel et al., 2017 [57], in vitro study
	AgNPs and hydroxyapatite was immobilized on titanium using plasma electrolytic oxidation and hydrothermal treatment.	Exhibited antibacterial and osteogenic activity.	Sobolev et al., 2019 [58], in vitro study
	A ceramic coating was established on the titanium surface using plasma electrolytic oxidation of nitrilotriiacetic acid (NTA)-based calcium-phosphate and AgNPs.	Exhibited antibacterial activity and high biocompatibility.	Oleshko et al., 2020 [59], in vitro study
	Silver nanoparticles immobilized on titanium implants using PEO.	Exhibited antibacterial activity. Cytotoxicity on SV-HFO cells was dependent on Ag concentration, the higher the concentration, the more cytotoxicity.	Necula et al., 2012 [60], in vitro study
Ti soaked in Ag solution with different reduction methods. (chemical reduction)	Titanium triple etched and immersed in tollens reagent (consists of a solution of silver nitrate, ammonia and some sodium hydroxide) to promote the formation of nano silver precipitations	Exhibited antibacterial activity and showed higher toxicity on NHOs when the concentration of silver was 0.1 ppm.	Pokrowiecki et al., 2017 [61], in vitro study
	Titanium surface was modified with citrate-capped AgNPs that spontaneously absorbed to the surface.	Exhibited inhibition of bacterial growth.	Flores et al., 2010 [62], in vitro study
hydrothermal treatment	Titanium surface was coated with Ag–Sr enriched nanofibrous titanium phosphate.	Exhibited antibacterial properties and improved cytocompatibility.	Garcia et al., 2021 [63], in vitro study
Electrodeposition	Different types of surfaces were evaluated: Machined + Ag, Shot-blasted with 20um Al_2_O_3_ particles + Ag, Shot-blasted with 60um Al_2_O_3_ particles + Ag	Exhibited no improvement on biofilm adhesion and cellular viability compared with the non-coated surface.	Vilarrasa et al., 2018 [64], in vitro study
	AgNPs deposited on titanium surface using Anodic Spark Deposition.	Exhibited antibacterial solid effect and optimal SAOS-2 cell adhesion and proliferation.	Della Valle et al., 2012 [65], in vitro study
Electroplating:	Titanium discs were plated with silver using the electroplating method, then the discs were coated with nHA or mHA using a sintering technique	Coating with Ag^+^ an nHA showed higher biocompatibility compared to AgNPs alone.	Salaie et al., 2020 [66], in vitro study
	Polished Ti-alloy discs with Ag and with a combination of nHA and mHA (hydroxyapatite) using the electroplating technique	Exhibited antibacterial activity and inhibited biofilm formation by 97.5%.	Besinis et al., 2017 [67], in vitro study
Hydrogel	Three-dimensional printed titanium alloy modified with hydrogel containing AgNPs. Clinical study on rabbit femurs.	In vitro experiments exhibited antibacterial activity, biocompatibility. Showed proliferation and osteogenic differentiation on BMSCs. In vivo experiments showed effective antibacterial properties and promoted bone regeneration.	Qiao et al., 2020 [68], in vitro study
	Titanium surface modified with hydrogel containing AgNPs.	Exhibited good antibacterial activity, no significant toxic effects on MG63 osteoblast like-cells.	De Giglio et al.,2013 [69], in vitro study
Microwave assisted synthesis	Nano Ag coating was applied with microwave-assisted synthesis. Clinical study on humans.	The coating suppressed the dental plaque adhesion on the healing abutments, no cytotoxic effect on human gingival fibroblasts.	Odatsu et al., 2020 [70], in vitro study
Sputtering technique	Titanium diss coated by pulsed magnetron-sputtering of nanocrystalline metals containing Ag.	The silver coating exhibited antibacterial activity and no cytotoxic effect on HaCaT cells.	Gosau et al., 206 [71], in vitro study
	Silver nanoparticles deposited on titanium abutments using direct current sputtering.	Exhibited good antibacterial effect; however, a high concentration of silver (6 µg/mm^2^) was required.	Kheur et al., 2017 [72], in vitro study
	Silver nanoparticles deposited on titanium sample using magnetron sputtering technique.	Exhibited antibacterial properties.	Lampe et al., 2019 [73], in vitro study

### 3.1. Surface Coating

**Figure 3:**
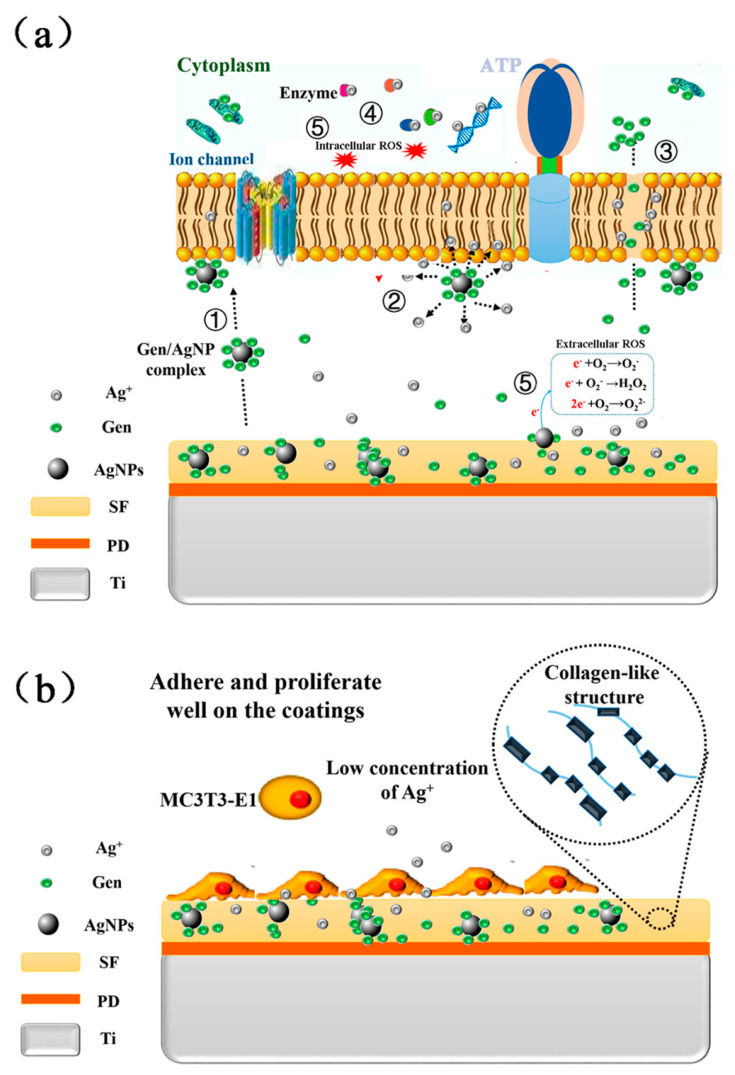
Schematic illustration of the possible antibacterial (**a**) and biocompatible (**b**) mechanism on the AgNPs/Gen-contained SF-based coatings. Reprinted with permission from [31]. Copyright 2017 American Chemical Society.

One approach to adding silver nanoparticles onto a titanium surface is to use a surface coating component. The examined studies that used this method showed improvement in the attachment of silver nanoparticles on the titanium surface and resulted in the stability of antibacterial properties. The surface coating components investigated include polydopamine, catechol functionalized chitosan, Phase-transited lysozyme with subsequent hyaluronic acid, chitosan, and chitosan-heparin polyelectrolyte multilayers, poly-L-lysine/Sodium alginate, hydroxyapatite/chitosan biopolymer, polysiloxane, TiO_2_/Ag containing copolymer and silicon oxide (Figure 3) [31,32,33,34,35,36,37,38,39,40]. These methods have shown moderate antibacterial activity against microbes shown in Table 2. One study showed that the antimicrobial activity against *E. coli* was lower than *S. Aureus* when catechol-functionalized chitosan was used as the linking component [32].

The surface coating component composition functions can be illustrated using polydopamine, which was used in several studies. Polydopamine (PDA) is an adhesive protein that is non-toxic and is used to improve the adhesion and biocompatibility of biomaterial surfaces [74,75,76,77,78]. Titanium surfaces coated with PDA and silver nanoparticles revealed a significant difference in bacterial growth rates of *S. mutans* and *P. gingivalis* compared to pure titanium, and the antibacterial effect remained the same for 48 h [36]. Several studies show that the duration of antibacterial activity increased when PDA-coating embedded with silver nanoparticles was used on titanium surfaces with a micro or nanostructure [79,80,81,82,83,84,85]. Albashari et al. used silver nanoparticles embedded in a polydopamine coating on a nanotubular titanium surface and examined the antibacterial effect against *P. gingivalis* using an MTT assay. The titanium samples with silver nanoparticles could still suppress the metabolic activity even after a seven-day pre-release. CCK-8 assay was used to examine the viability and proliferation, and alkaline phosphatase activity was used to examine the osteogenic differentiation of dental pulp stem cells. This study showed that PDA promotes better attachment of the silver nanoparticles and, therefore, better biocompatibility and osteogenic capacity than titanium nanotubes coated directly with silver nanoparticles. However, the combination of TNT/PDA/Ag and bFGF(Basic fibroblast growth factor) promoted the highest cell viability, proliferation and osteogenic capacity. In an in vitro study, AgNPs/Gentamycin embedded silk-fibroin coating linked to the titanium surface by a PDA layer achieved a synergetic antimicrobial effect when combining silver nanoparticles with gentamycin. In addition, silk fibroin promoted additional proliferation and osteogenic capacity [31].

### 3.2. Glass Containing Silver Nanoparticles on Titanium Surface

**Figure 4:**
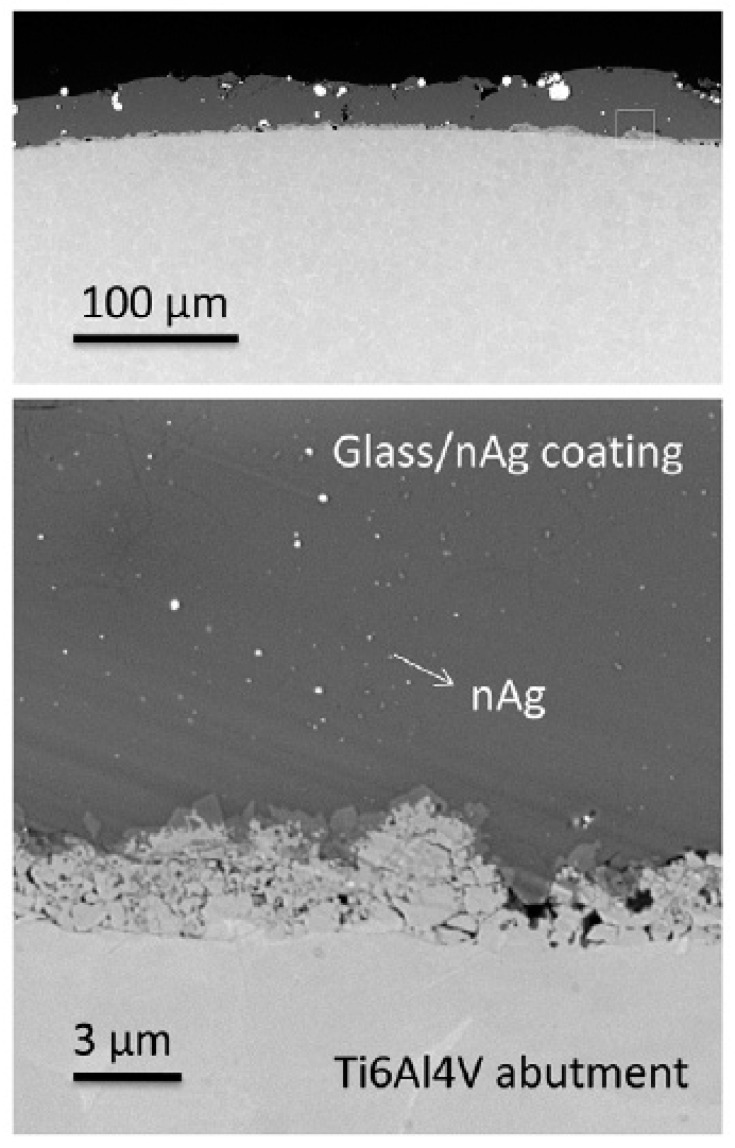
Scanning electron micrographs at different magnifications of a cross-section of the abutment. Reprinted with permission from [44] under a Creative Commons License.

Another method of incorporating silver nanoparticles into the titanium surface is by coating the implant surface with soda-lime glass in which silver nanoparticles are embedded (Figure 4). The soda-lime glass was used to achieve even dispersion of the silver nanoparticles, prevent agglomeration, and control and sustain silver nanoparticle delivery [41,43,44]. An in vitro study performed by Cabal et al. showed that titanium coated with glass containing AgNPs significantly differed in biofilm mass and bacterial adhesion of *Streptococcus oralis* compared to uncoated titanium and titanium coated with glass [41]. The concentration of silver release was found to be below the cytotoxic level for human fibroblasts, as shown in previous studies [86], and the release profile indicates a controlled silver release for at least seven days. In vivo studies have shown that additional bone loss after clinically induced peri-implantitis was three times greater in control samples than in the glass containing silver nanoparticle-coated implants [44]. Martinez et al. [43] also confirmed reduced bone loss when using silver-containing coating compared to the control in an in vivo study. This study also examined the dimensional coating changes within the mouth with prolonged use. It showed significant changes in the coating thickness from an initial mean thickness of 51 µm to 44 µm on the buccal side and 26 µm on the lingual side after three months of use. In addition, defects and cracks could also be seen, giving uncertainty regarding the long-term cytotoxic effect, and the accumulation of particles in the organism in addition to the mechanical properties of the implant. However, these studies conclude that this method is promising for bone loss reduction in experimentally induced peri-implantitis.

### 3.3. Silver Nanoparticle Applied on Micro/Nano Titanium Surfaces

#### Nanotubes

**Figure 5:**
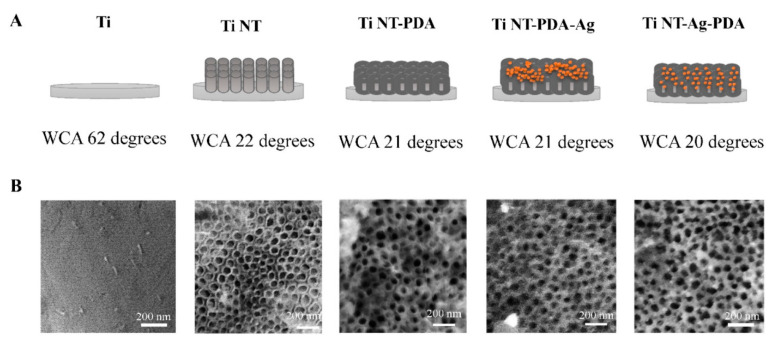
Physicochemical characterization of different, AgNP loaded nanotubular titanium surfaces. (**A**) schematics of the different nanotubular Ti surfaces and their water contact angles (WCA); (**B**) SEM micrographs of the different titanium surfaces with and without nanotubules. The bright spots in the SEM of Ti NTPDA-Ag represent AgNPs. Reprinted with permission from [50] under a Creative Commons license.

Nanotubes refer to a tubular surface structure within the nanometer range that can be synthesized using different surface modification techniques such as anodization, plasma electrolytic oxidation, and alkali hydrothermal reaction. This increases the roughness and surface area, promoting better osseointegration; however, this creates more favourable conditions for bacterial growth (Figure 5). Silver nanoparticles were added to Ti-nanotubes to prevent bacterial growth and biofilm formation [45,46,47,48,49,50,51,52,53]. Titanium nanotubes filled with AgNPs showed good antibacterial activity against Gram-positive and Gram-negative bacteria shown in Table 3.

Silver nanoparticles penetrate the nanotubular structure, resulting in a slower release of silver ions, which provides a long-lasting antibacterial capability and less cytotoxic silver concentration. Different techniques have been used to deposit silver on Ti-nanotubes. A study by Wang et al. compared titanium nanotubes filled with silver ions with metallic nanoparticles [45]. Both discs showed the same bactericidal rate (99.99%). However, there was no significant difference between the inhibition zone diameters in both samples.

There was a significant difference in the longevity of the antimicrobial capacity. The antibacterial properties of AgNP-filled nanotubes extended for more than 15 days because of the slow release of silver nanoparticles. The concentration of released silver ions after 15 days was 50 ppb, which is still above the critical concentration and 0.1 ppb of antibacterial effect as shown in previous studies [87,88]. In contrast, the silver ion-containing nanotubes had a significant silver ion release in the first five days, depleted on the 7th day, and ultimately losttheir antibacterial effect, leading to significant cell toxicity. The AgNP filled NTs, however, showed excellent cytocompatibility and cell proliferation. In a study performed by Cheng et al., the increased diameter of the nanotubes resulted in higher expression of ALP, meaning better osteogenic activity. This study also included an in vivo experiment on rats that exhibited more new bone formation at the defect zone and almost complete healing six weeks after surgery using strontium (Sr) and a silver-loaded nanotubes (NT) (NT_40_-Ag_1.5_Sr_3)._ However, the bone formation was less pronounced for the samples with a higher silver concentration (NT_40_-Ag_2.0_Sr_3_), whereas simple titanium and unfilled TiO_2_-nanotubes resulted in limited bone healing [47]. These studies demonstrated that a nanotubular titanium structure with silver nanoparticle deposition displays long-lasting antibacterial properties, high osteogenic potential, and improved cytocompatibility.

### 3.4. SLA—Treated Surfaces with Silver—Plasma Immersion Ion Implantation (AgPIII)

**Figure 6:**
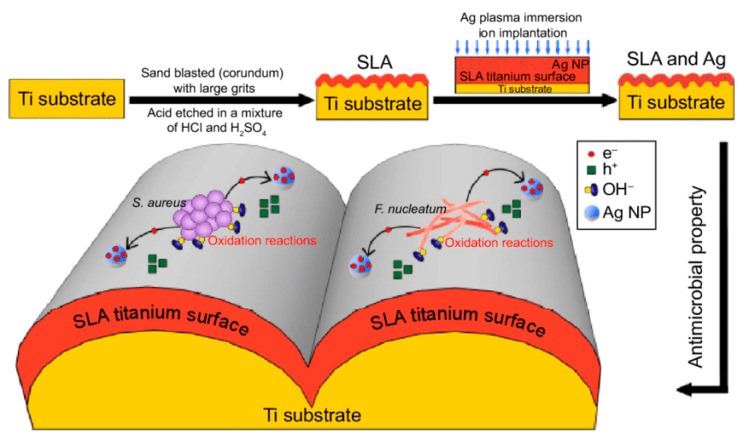
Schematic diagram of the fabrication procedures for the micro/nanostructured titanium and illustration for the possible antibacterial mechanism of the Ag NPs immobilized on the SLA-produced titanium oxide layer. Notes: 30 min-15 Ag-PIII, titanium surfaces treated by first SLA procedure and then silver plasma immersion ion implantation at 15 kV for 30 min; 60 min-15 Ag-PIII, titanium surfaces treated by first SLA procedure and then silver plasma immersion ion implantation at 15 kV for 60 min; 90 min-15 Ag-PIII, titanium surfaces treated by first SLA procedure and then silver plasma immersion ion implantation at 15 kV for 90 min. Abbreviations: Ag NPs, silver nanoparticles; SLA, sandblasted, large grit, and acid-etched; Ag-PIII, silver plasma immersion ion implantation; min, minutes. Reprinted with permission from [55] under Creative Common licence.

SLA (Sandblasted large grit acid etched) surface modification has been used to create microtopography, and Ag-PIII was used to immobilize silver nanoparticles on the titanium surface, which created a nanotopography (Figure 6). This results in a hierarchical micro/nanostructured surface that mimics the hierarchical structure of the natural extracellular matrix and bone tissue. In vitro studies show that this surface modification results in better osseointegration by actively stimulating bone cell differentiation and protein production [23,55,56].

The study by Zhu et al. used different tests to evaluate the antibacterial effect against the Gram-negative bacteria, *Fusobacterium nucleatum*, and the Gram-positive bacteria, *Staphylococcus aureus*. The tests include antimicrobial assays by fluorescence staining, spread plate method, and SEM observation. These tests were performed on three different SLA-treated samples modified with Ag-PIII at 15 kV for 30, 60, and 90 min, respectively. The resulting antimicrobial properties were ranked in the following order, 90 min-15 AgPIII > 60 min-15 AgPIII > 30 min-15AgPIII. The spread plate method showed significant differences in the antimicrobial activity between the three samples. The antimicrobial activity against *F. nucleatum* was superior compared to *S. aureus*. The reduction rates of *F.nucleatum* were ∼92%, 57% and 22%, whereas 80%, 42% and 12% for *S. aureus*. However, there were no significant differences in antibacterial activity after 30 days of incubation in PBS or when longevity and stability were tested. These results indicate that the AgPIII treated samples were stable with a long-lasting antimicrobial effect. MTT-assay and live/dead staining indicated no apparent cytotoxicity, and the proliferation and viability of rBMSCs was also promoted. AgPIII did not prevent osteoblastic differentiation [55].

Qiao et al. performed an in vivo study using Labrador dogs to evaluate bone apposition around SLA-modified AgPIII treated titanium implants. Three different SLA treated samples were modified with Ag-PIII at 30 kV for 30, 60 and 90 min, respectively. Implant stability, Micro-CT evaluation and histological analysis examined implant properties. All AgPIII samples achieved good primary stability and no clinical mobility. After eight weeks, AgPIII showed significantly higher stability than the control group; however, after 12 weeks, this difference was reduced. Micro-CT evaluation showed that the 30 min-30 AgPIII and 60 min-30 AgPIII samples had significantly higher bone volume (BV), bone mineral density (BMD), BV/total volume, trabecular thickness and number compared to the 90 min-30 AgPIII and the control samples. No significant differences were found between the 30 min-30 AgPIII and 60 min-30 AgPIII samples. Histological analysis showed direct contact between the connective tissue and implant surface, creating a soft tissue seal between the oral environment and the bone. The histomorphometric measurements (percentage of BIC and BDWT ratio) confirmed the micro-CT evaluation where the 30 min-30 AgPIII and 60 min-30 AgPIII samples showed significantly better results than the 90 min-30AgPIII and the control samples [23].

Plasma immersion ion implantation has also been used to add other metallic nanoparticles on the implant surfaces to achieve antibacterial activity (Figure 6), such as Zn. For example, a study performed by Jin et al. used PIII to combine Zn and Ag nanoparticles on a polished implant surface, which concluded that Ag nanoparticles alone result in more cytotoxicity. At the same time, combining Zn and Ag can achieve more cell proliferation and antimicrobial properties [89].

Other in vitro studies have used different methods to add Ag nanoparticles on titanium surfaces, such as electrodeposition, electroplating, sputtering technique, etc. These studies generally revealed good antibacterial and cytotoxic effects. However, they are not supported by enough evidence [54,64,65,66,67,68,69,71,72,73].

## 4. Discussion

Implant-related infections such as peri-implant mucositis and peri-implantitis are common complications in the dental field; clinicians have no agreement about the best treatment option [90]. Implant-based factors, such as surface characteristics, might increase the risk of developing peri-implant diseases [91]. Hence, several studies have aimed at developing titanium surfaces with antibacterial properties. One of these methods includes using silver nanoparticles. Silver has been shown to have a broad spectrum of antibacterial activity accomplished by disrupting the bacterial cell wall permeability, DNA damage and inactivation of essential proteins.

The most concerning aspect of using silver is the potential adverse effects. Numerous researchers have shown that some MNPs can induce decreased cell survival rate, production of reactive oxygen species (ROS), mitochondrial damage, DNA strand breaks, and even autophagy, pyroptosis, apoptosis, or other forms of cell death [22,92]. In addition, impairment of osseointegration must be avoided. However, different methods have been developed to satisfy these requirements.

The included studies have examined the antibacterial activity against different bacterial species. In general, they achieve satisfactory results regarding the antibacterial effect. However, some studies use bacterial species that are not considered to contribute to the biofilm formation required to initiate peri-implant infections, and even bacteria rarely found in the oral cavity. Bacteria such as *E. coli* and *S. aureus* are not that common in the oral cavity and have been used by several studies for evaluating the antibacterial effect [45,47,48,50,51,52]. Thus, it is not sure that the results from these articles can be applied to the oral cavity. Furthermore, several studies did not perform antibacterial tests on a biofilm with a higher tolerance against antibacterial and antiseptic agents. Based on this, it is impossible to conclude if the antibacterial activity found in these studies can be transferred to the oral cavity.

The included studies performed different biocompatibility tests on the cell cultures of animals or humans. Most studies investigated cytotoxicity using MTT-assays and osteogenic differentiation by measuring ALP activity. They found convenient cytotoxicity when the silver nanoparticles were added to the titanium surface using a polymer as surface coating and/or when a micro/nano-structured surface such as nanotubes was used. These techniques created a better attachment and reduced mobility of silver nanoparticles on titanium surfaces. Since fewer silver ions were released, its cytotoxic impact on cells was lower, which may give implants increased longevity. Few in vivo studies on animals examined the cytotoxic effects and biocompatibility of the silver nanoparticles. The systemic effects of silver nanoparticles have been evaluated. However, the systemic adverse effects of silver nanoparticles on dental implants have not been performed clinically. It is known that silver-containing wound dressings on the skin may result in discolouration; it is unknown whether the same will apply to the oral mucosa by using silver-coated titanium implants. However, if this is the case, silver particles on a dental implant may cause aesthetic issues due to possible miscolouring of the mucosa. According to the current literature, silver nanoparticle-coated dental implants’ clinical adverse effects are less investigated. Therefore, it is impossible to conclude whether these techniques can achieve a biocompatible and acceptable outcome for dental implants. Substantial consideration is devoted to the design and validation of dental materials, and their interactions with metal ion release to peri-implant tissues are commonly neglected. There is a gap of knowledge in our understanding of designing dental materials with optimal sliver physicochemical properties and/or poor stability of biomaterial properties after implantation in the jaw. The success of host responses to dental materials or biomaterials depends on its chemical state regulating the body’s cell signalling pathways [93].

A gradual reduction of silver concentration from the implant surface occurred over time in all the included studies. The maximal duration to examine the silver release and the antimicrobial effect was 15 days. Even though this study exhibited good results within 15 days, this only reflects a short-term effect. At some point, the concentration of silver on the titanium surface will become so low that it loses its antibacterial properties. Therefore, confirming that these application methods will give a long-term antimicrobial effect is impossible, and long-term protection against peri-implant infections cannot be expected. A recent study showed that the release of metal particles and ions from dental implants is pro-inflammatory and causes infiltration of inflammation. Such debris has cytotoxic and genotoxic potential for adjacent tissues. Thus, the amount and physicochemical properties of the nanoparticle releases determine the magnitude of the harmful consequences to peri-implant tissues [94].

The in vitro and in vivo studies used in this review showed variation in experimental set-ups, quality and quantity. This made the comparison between the different strategies difficult. In addition, it was difficult to determine the most promising method. Among the limitations of the included studies is that they did not recreate the conditions in the oral cavity. Most in vitro studies examined the antibacterial effects on monocultures cultured under static growth conditions. However, the oral cavity has diverse bacterial flora. In the oral cavity, bacterial adhesion and biofilm formation are influenced by numerous factors, such as pellicle formation, host immune system and adhesins. Another limitation is the use of varying antimicrobial tests among the included studies.

## 5. Conclusions

Most of the reviewed studies revealed that silver nanoparticles on dental implant surfaces reduced cytotoxicity and provided a prolonged antibacterial effect. The cytotoxicity and antibacterial effect are closely linked to how the silver nanoparticles are released from the titanium surfaces, where a slower release increases cell viability and proliferation. Few clinical studies with silver nanoparticles studies are available. Therefore, the clinical benefits cannot be stated. In addition, there are concerns regarding biocompatibility and possible miscolouring of the mucosa. However, more in vivo and clinical studies are required to address those issues adequately in addition to the systemic effects. Since peri-implantitis is a complex disease, any treatments, such as silver nanoparticles, need to be validated clinically before one judges its performance. The lack of such validation is a limiting factor. Future studies should focus on the adverse reactions of the peri-implant tissues by providing a safer and more reliable release of Ag nanoparticles. This could lead to more clinical translation of such Ag-nanoparticle surfaces, as none have been tested clinically.

## Figures and Tables

**Table 2:** Polymeric surface coating with embedded silver nanoparticles onto a titanium surface has successfully reduced the Gram-positive and Gram-negative bacteria.

Silver Nanoparticles on Titanium Surface Effect on Various Bacteria	
Gram-Positive Bacteria	Gram-Negative Bacteria
*Staphylococcus aureus* [31,32,33,35,37,39] *Streptococcus mutans* [34,36,37] *Streptococcus sanguinis* [34] *Staphylococcus epidermidis* [35] *Streptococcus salivarius* [38]	*E. coli* [32] *Aggregatibacter- actinomycetemcomitans* [34] *Porphyromonas gingivalis* [36,40]

**Table 3:** Titanium nanotubes filled with AgNPs showed appropriate antibacterial activity against Gram-positive and Gram-negative bacteria.

Titanium Nanotubes Filled with AgNPs Effect on Various Bacteria Species	
Gram-Positive Bacteria	Gram-Negative Bacteria
*Staphylococcus aureus* [47,48,50,51,52] *Streptococcus mutans* [49] *Staphylococcus epidermidis methicillin-resistant Staphylococcus aureus* [47]	*Escherichia coli* [45,47] *P. gingivalis* [46] *Pseudomonas aeroginosa* [50]

## Data Availability

Not applicable.
